# Does Prenatal Exposure to Maternal Inflammation Causes Sex Differences in Schizophrenia-Related Behavioral Outcomes in Adult Rats?

**DOI:** 10.1523/ENEURO.0393-19.2019

**Published:** 2019-10-15

**Authors:** Rosalind S.E. Carney

## Abstract

**Highlighted Research Paper:** Maternal Immune Activation during Pregnancy Alters the Behavior Profile of Female Offspring of Sprague Dawley Rats, by Brittney R. Lins, Wendie N. Marks, Nadine K. Zabder, Quentin Greba, and John G. Howland

*The fetal origins of adult disease hypothesis* (also known as the Barker hypothesis) posits that prenatal adversity can result in chronic conditions that manifest during adulthood (Barker, 1990). For example, low birth weight is associated with increased risk for coronary artery disease, type 2 diabetes, and neurological disorders (Calkins and Devaskar, 2011). Several ecological studies have reported that influenza infection during pregnancy (maternal infection) increases the risk for the development of schizophrenia in offspring as adults (Watson et al., 1984; Barr et al., 1990; Takei et al., 1993). In a Finnish birth cohort of the 1957 type A2 influenza epidemic, the highest risk for schizophrenia in prenatally exposed offspring occurred during the second trimester (Mednick et al., 1988). Later studies associated influenza infection during the second trimester with a threefold to sevenfold increase in risk (Brown and Susser, 2002; Brown et al., 2004; Brown, 2006).

Animal models provide an expedient means to examine neurological disorders that have an adult onset but stem from prenatal pathophysiological insults (Howland et al., 2019). In rodents, the infection of pregnant dams with human influenza virus results in the adult onset of schizophrenia-related cognitive and behavioral deficits that can be traced back to developmental abnormalities first evident in the fetal or early postnatal brain. For example, reductions in the number of dopaminergic neurons in the midbrain or in dopamine receptor expression levels hamper the normal development of dopaminergic circuitry in mice (Vuillermot et al., 2010). Therefore, it is important to examine in detail any associations that can be found between the physiological consequences of maternal infection, in both the mother and developing fetus, and the pathophysiological events that increase susceptibility for schizophrenia in offspring.

The systemic inflammation that occurs during maternal infection has been linked to an increased risk for psychiatric illnesses in the offspring (Patterson, 2011; Jiang et al., 2016; Brown and Meyer, 2018; Gustafsson et al., 2018). During maternal infection, levels of inflammatory cytokines are elevated in the maternal blood circulation and placenta; direct or indirect exposure could affect the developing fetal brain (Boksa, 2008). Maternal immune activation (MIA) describes the systemic maternal inflammatory phenotype that can be induced in pregnant rodents using the immunostimulant polyinosinic:polycytidylic acid (polyI:C), a synthetic agonist of the Toll-like receptor 3. In a prior *eNeuro* publication, Lins et al. (2018) examined whether maternal serum cytokine levels following polyI:C-induced MIA could act as a predictor for adult cognitive defects related to schizophrenia in male offspring. In the current *eNeuro* publication, Lins et al. (2019) present the findings from the female siblings of the male cohort examined in the prior study.

On gestational day (GD) 15, timed-pregnant rat dams were anesthetized and injected with 0.9% saline or polyI:C (4 mg/kg) via the tail vein. Three hours later, the dams were anesthetized again to facilitate the drawing of a blood sample from the tail vein, contralateral to the vein used for saline or polyI:C injection. The blood sample was used to determine whether the maternal serum cytokine [chemokine ligand 1 (CXCL1), IL-6, CXCL2, and TNF-α] levels could be correlated with any behavioral defects in the adult offspring. Estrous phase was determined by vaginal cytology to determine whether behavioral outcomes were influenced by reproductive hormones. The polyI:C-injected pregnant dams exhibited a short-term reduction in body weight, and an elevation in CXCL1 and IL-6 levels, but no change in CXCL2 or TNF-α levels.

The battery of behavioral tests related to positive, negative, and cognitive symptoms of schizophrenia was performed in the following order: prepulse inhibition (PPI), cross-modal object recognition (CMOR), sociability, oddity discrimination, and MK-801-induced locomotor activity (Fig. 1). All female offspring were used for the PPI test, whereas one to two female offspring were used for all other tests; results were averaged between females of the same litter. Each behavioral test is briefly described, and the findings in adult females are presented and compared with those of the male siblings reported in the prior study (Lins et al., 2018).

**Figure 1.**
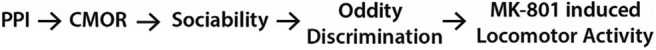
Flowchart depicting the order of the battery of behavioral tests (Adapted from Figure 1 in Lins et al., 2019.).

PPI is used as a measure of sensorimotor gating of the startle reflex response, which is disrupted in schizophrenia (Mena et al., 2016). When a brief, relatively quiet prepulse tone preceded a 120 dB startling tone, the percentage of attenuation to the startling tone was measured in polyI:C-exposed and control offspring. No treatment effect was found for prenatal polyI:C exposure on PPI in either male or female offspring. However, male prenatally polyI:C-exposed adult offspring showed a heightened startle response to the 120 dB tone alone (Lins et al., 2018).

CMOR is a spontaneous exploratory behavioral test that relates to cognitive impairments in schizophrenia. Exploration of objects in the arm of a Y maze was first assessed during a tactile test under red light conditions, impairing visual assessment. A visual test was also conducted under white light conditions where rats were restricted to visual observation of the objects as Plexiglas barriers were installed in the Y maze to prevent the use of tactile recognition. The cross-modal phase included both a tactile sample phase and a visual test phase. Spending more time exploring a novel object than a familiar object during the test phase indicates that memory recognition occurred. Neither polyI:C-exposed nor control female offspring exhibited cross-modal memory (Fig. 2). In the male sibling cohort, a cross-modal memory deficit was only observed in the polyI:C-exposed offspring (Lins et al., 2018).

**Figure 2.**
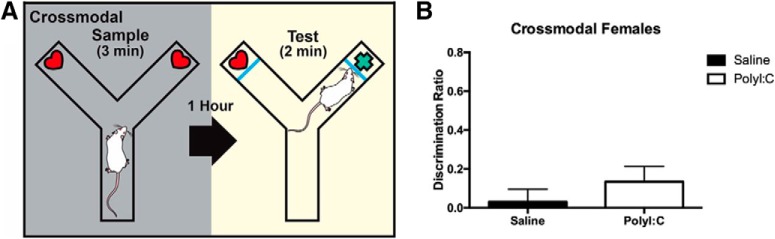
Experimental setup and results of cross-modal recognition task. ***A***, The Y maze assembled for the cross-modal phase, which has a tactile sample phase and visual test phase. ***B***, Adult offspring prenatally exposed to polyI:C or saline failed to display cross-modal recognition memory as novel object exploration was equal to chance. Schematic has been published previously (Lins et al., 2018; Paylor et al., 2016) (Adapted from Figure 4 in Lins et al., 2019.).

The sociability test relates to the social withdrawal aspect of schizophrenia. When presented with a stranger conspecific, prenatal polyl:C exposure resulted in sociability deficits in females, compared with control offspring. The male siblings also demonstrated reduced sociability (Lins et al., 2018).

The oddity discrimination task involved a white square arena that contained an object in each corner; three objects were identical, one was unique (odd). The percentage time of odd object exploration was calculated with respect to the total object exploration time. While control offspring preferentially explored the odd object, prenatal polyI:C exposure impaired this discrimination, despite equivalent total exploration time (Fig. 3). Oddity discrimination was also impaired in the cohort of male siblings (Lins et al., 2018).

**Figure 3.**
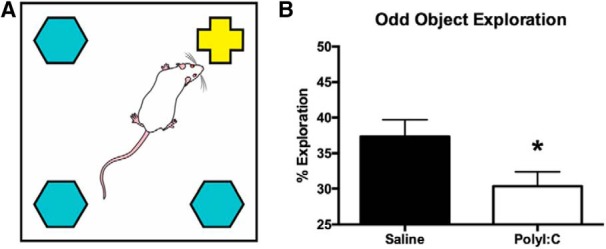
Experimental setup and results of the oddity discrimination task. ***A***, Schematic of the white square arena used to conduct the oddity discrimination task showing the arrangement of three identical objects and one different, or odd, object. The schematic has been published previously (Lins et al., 2018). ***B***, Bar graph displaying the percentage of total object exploration spent examining the odd object. Prenatally polyI:C-exposed adult offspring displayed significantly less oddity preference than offspring exposed to saline prenatally (*p < 0.05). (Figure 5 from Lins et al., 2019.).

The MK-801-induced locomotor activity test relates to the increased hyperactivity and elevated striatal dopamine levels observed in a subset of individuals with schizophrenia (Powell and Miyakawa, 2006). MK-801 is an NMDA receptor antagonist that induces locomotor hyperactivity. Both saline- and polyI:C-exposed females showed increased locomotor activity in response to a 0.1 mg/kg dose of MK-801. Male siblings that were exposed to polyI:C prenatally exhibited heightened locomotor activity compared with controls, but MK-801 was used at a dose of 0.2 mg/kg (Lins et al., 2018). A lower dose of MK-801 was used for the female cohort to enable better comparison with prior studies (Andiné et al., 1999; Howland et al., 2012; Zhao et al., 2013).

Overall, maternal cytokine levels were not predictive of adverse neurological outcomes in adult offspring, although it is possible that a relationship could be found for other cytokines. Nonetheless, MIA was correlated with similar or sex-specific behavioral or cognitive deficits in the female and male adult offspring. Potentially, more severe neurological phenotypes could arise if the timing of MIA was adjusted because MIA activates the maternal immune system during a restricted time window (Brown and Meyer, 2018). MIA at GD 12.5 in mice caused adult behavioral changes that were not observed in the offspring of pregnant dams that were injected with an anti-IL-6 antibody at the time of MIA induction (Smith et al., 2007). A prior study found that neurobehavioral deficits in offspring were influenced by whether MIA induced weight loss in the pregnant dam or not (Missault et al., 2014). Therefore, many variables may underlie the variation in behavioral outcomes following prenatal adversity, and guidelines to improve the reproducibility of MIA studies have been published recently (Kentner et al., 2019).

It is not known to what extent schizophrenia-related symptoms in adult offspring prenatally exposed to maternal infection with influenza are caused by factors pertinent to the offspring alone, such as stress, sleep deprivation, or medications (Patterson, 2002). The *eNeuro* publications by Lins et al. (2018, 2019) stress the importance of studying both sexes in outcomes related to psychiatric illnesses. Neuroscience research has tended to heavily focus on male subjects despite mandates from the National Institutes of Health and the Canadian Institutes of Health Research to include female subjects (Clayton, 2018; Shansky, 2019). A study of neuroscience publications from 2010 to 2014 showed that, while more publications detailed the sexes used, the sex bias toward the preference of male subjects still existed (Will et al., 2017). In MIA studies, published in 2000–2018, 44.5% and 3.4% of studies examined only male or female offspring, respectively (Coiro and Pollak, 2019). The *eNeuro* publications by Lins et al. (2018, 2019) show that subtle differences in behavioral and cognitive outcomes exist between the sexes. Sex differences in the age of onset of schizophrenia also provide strong support for the consideration of sex as a significant factor in experimental design (Ochoa et al., 2012). A serious consequence of excluding female subjects in scientific research is the development of medications that ignore aspects of female reproductive physiology, such as menstruation and ovulation (Galea, 2019).

Ongoing and future studies in Professor John Howland’s laboratory (University of Saskatchewan, Saskatoon, Saskatchewan, Canada) aim to develop “multi-hit” models of *in utero* insults that produce more severe neurological phenotypes in adult offspring. Recently, another research group (Lacaille et al., 2019) showed that combining lipopolysaccharide-induced MIA with neonatal chronic sublethal hypoxia produced a better mouse model of the neurological consequences of premature birth than either insult alone. Howland and colleagues (2012, 2019) are also investigating mechanisms to reduce MIA by blocking inflammatory signaling pathways during gestation in rats. Howland and colleagues (2012, 2019) have previously demonstrated that MIA causes alterations in extracellular matrix structures, termed perineuronal nets, in the medial prefrontal cortex in particular (Paylor et al., 2016).
